# Impacts of Whole-Grain Soft Red, Whole-Grain Soft White, and Refined Soft White Wheat Flour Crackers on Gastrointestinal Inflammation and the Gut Microbiota of Adult Humans

**DOI:** 10.3390/biology13090677

**Published:** 2024-08-30

**Authors:** Gigi A. Kinney, Eliot N. Haddad, Neha Gopalakrishnan, Kameron Y. Sugino, Linda S. Garrow, Perry K. W. Ng, Sarah S. Comstock

**Affiliations:** Department of Food Science and Human Nutrition, Michigan State University, East Lansing, MI 48824, USA

**Keywords:** fiber, microbiota, whole grain, wheat, butyrate, calprotectin, lipocalin-2

## Abstract

**Simple Summary:**

Our understanding of methods by which dietary interventions can be used to modify the established gut microbiota in adult humans is rudimentary. In this intervention trial, a person’s gut microbial diversity and intestinal inflammatory markers remained unchanged across four weeks of daily consumption of 80 g of wheat crackers, regardless of wheat-flour type. Consumers must understand that shifting their gut microbiota and inflammatory state with a single dietary constituent may be difficult with mild and short-term interventions.

**Abstract:**

Consumption of whole-grain wheat has been associated with positive health outcomes, but it remains unclear whether different types of wheat elicit varying effects on the gut microbiome and intestinal inflammation. The objectives of this research were to investigate the effect of two whole-grain wheat flours versus refined wheat flour on the diversity of the human gut microbiota, as well as on butyrate production capacity and gastrointestinal inflammation, using one-week dietary interventions. For this study, 28 participants were recruited, with ages ranging from 18 to 55 years and a mean BMI of 26.0 kg/m^2^. For four weeks, participants were provided 80 g daily servings of different wheat crackers: Week A was a run-in period of crackers made from soft white wheat flour, Week B crackers were whole-grain soft white wheat flour, Week C crackers were a wash-out period identical to Week A, and Week D crackers were whole-grain soft red wheat flour. At the end of each week, participants provided fecal samples that were analyzed for markers of intestinal inflammation, including lipocalin and calprotectin, using enzyme-linked immunosorbent assays and quantitative real-time PCR. The primary outcome, gut bacterial community alpha and beta diversity, was similar across timepoints. Three taxa significantly differed in abundance following both whole-grain wheat flour interventions: *Escherichia/Shigella* and *Acidaminococcus* were significantly depleted, and *Lachnospiraceae NK4A136* group was enriched. Secondary outcomes determined that protein markers of intestinal inflammation and genes related to putative butyrate production capacity were similar throughout the study period, with no significant changes. Lipocalin concentrations ranged from 14.8 to 22.6 ng/mL while calprotectin ranged from 33.2 to 62.5 ng/mL across all 4 weeks. The addition of wheat crackers to the adult human subjects’ usual diet had a minimal impact on their gastrointestinal inflammation or the gut microbiota.

## 1. Introduction

The gut microbiota is a community of microbes that impacts the biological, immunological, and metabolic functions of the host. In the mature adult gut, a diverse microbiome is associated with positive health outcomes such as improved glucose regulation, lower levels of inflammation, and decreased weight gain [1,2]. Furthermore, interruptions to the homeostasis of the gut microbiota have been associated with inflammatory bowel disease, among other gastrointestinal diseases, obesity, metabolic syndrome, diabetes, and a range of other diseases [3,4].

Diet is an important factor shaping the gut microbiome [5]. In the first few months of life, the transition from human milk/formula to solid foods instigates the shift from an infantile to an adult-like gut microbiota [6]. Later in life, specific foods and nutrients modify the microbiome, and there is evidence that these changes subsequently impact health [3]. Increased consumption of dietary fiber has been linked with increased microbial richness and stability [7]. With the increasing global adoption of the Western diet, characterized by energy-dense and heavily processed foods, there has been a concurrent shift towards gut microbial dysbiosis and a rise in maladies such as diabetes, cardiovascular diseases, gastrointestinal diseases, and atopy [8]. Thus, the Western diet, as a function of industrialization, may be predisposing individuals to numerous chronic diseases through alterations to the gut microbiota [9].

Whole grains are rich in fermentable fibers that are accessible to the gut microbiota for food and energy [10] and also confer health benefits [11]. The total dietary fiber content of whole-grain wheat ranges from 9 to 20% on a dry-weight basis [12]. Thus, these fibers may modulate the health of the host by modifying the gut microbiota. Fiber intake contributes to the global variability in gut microbial diversity, as illustrated by the difference in gastrointestinal microbial communities between South American populations consuming a diet full of plant-based carbohydrates and North American populations consuming a low-fiber diet [13]. Dietary intervention studies examining the relationship between whole-grain intake and gut microbiota are numerous. In rats, whole rye intake over a 12-week period increased fecal microbial diversity and decreased the Firmicutes/Bacteroidetes ratio compared to those that consumed refined rye [14]. In a human intervention study, adding 60 g of whole-grain barley and/or brown rice to the diet for four-weeks increased microbial diversity [15]. In a separate intervention study, habitual low consumers of whole grains consumed a diet rich in whole grains and experienced no change in the fecal microbiota after the 6-week intervention [16]. Emerging work also suggests that the beneficial health effects of whole-grain wheat consumption may be due to potential modulatory effects on inflammation mediated by the gut microbiota [17]. In unhealthy individuals, whole-grain interventions have a reproducible anti-inflammatory effect, but studies in healthy individuals have led to mixed results. Thus, though there are numerous studies examining the relationship between whole-grain intake and gut microbiota, the effects of whole-grain wheat on the overall diversity and composition of the human gut microbiota have been largely inconclusive. Hence, more research is needed to understand how specific types of whole grains influence the microbiome and how these perturbations impact health.

Types of wheat grains (red, white, soft, hard, spring, and winter) vary in their composition of phytochemicals, micronutrients, and fiber [18]. These compositional differences potentially underlie unique effects on the gut microbiota [19]. White whole wheat is higher in bound phenolics, which may be more likely to survive digestion and impact the intestinal microflora [18]. From a sensory standpoint, white whole wheat is preferred among consumers as opposed to red whole wheat, although darker wheats such as red whole wheat are perceived as healthier. Whole white wheat has also been deemed to be sweeter tasting, and whole red wheat has been described as being bitter tasting. Therefore, it is important to consider consumer acceptance, as well as the type of grain being consumed. There is also an underwhelming amount of research to elucidate the effects of different wheat types in commonly consumed amounts, about 45–100 g [20] or one ounce equivalent of whole grains [21], on the gut microbiota. Hence, we sought to clarify the relationship between wheat and the gut microbiota in a sample of free-living adult humans, with a specific focus on understanding the potential differences in effects derived from whole-grain soft red (WGSR) wheat flour versus whole-grain soft white (WGSW) wheat flour versus refined soft white (RSW) wheat flour. Furthermore, each type of cracker was consumed for one week in order to evaluate if such a period of time was adequate to induce rapid changes in the gut microbiome. In one study, human adults that were fed animal-based diets with high amounts of meats, cheese, and eggs showed significant increases in beta diversity (q < 0.05) only one day into diet intervention [22]. Additionally, most dietary intervention studies record and collect data for 3–7 days, and 7 days has been regarded as sufficient time to modify the gut microbiome with diet in healthy adults [23]. Hence, through this study, another aim was to assess whether observable results could be produced in a weeklong dietary intervention of whole-wheat crackers.

Here, we used a population of 28 adults to investigate the effect of two different types of whole-grain wheat flours versus refined flour on the gut microbiota, analyzing changes in alpha and beta diversity, as well as in butyrate production capacity and gastrointestinal inflammation. We hypothesized that each of the whole-grain-flour types would increase microbial diversity and decrease intestinal inflammation compared to the refined wheat flour type. Studies in healthy individuals have led to mixed results regarding the anti-inflammatory effects of whole grain; thus, we examined whether markers of intestinal inflammation were significantly altered by the type of wheat flour consumed.

## 2. Materials and Methods

### 2.1. Design

The design of this study was previously described in depth [24]. In short, this four-week study provided participants with crackers made from three different wheat flour types. Participants were instructed to follow their normal diet, while incorporating 80 g of the provided wheat flour crackers into each day. Cracker ingredients included flour (approximately 13% moisture), iodized salt, sugar, vegetable shortening, and tap water (Table 1). The RSW crackers contained about 4 g of fiber per daily serving, whereas the whole-grain crackers contained about 11 g of fiber per daily serving. Each week, the participants would receive crackers made of a different type of wheat flour. In Week A, participants daily consumed crackers made of RSW wheat flour as the run-in period. In Week B, crackers were made of WGSW wheat flour. In Week C, a washout of the same crackers as Week A was provided to participants. Finally, participants consumed crackers made of WGSR wheat flour in Week D. Stool samples were collected into plastic commode hats (Fisher Scientific, Waltham, MA, USA) by the participants in their homes during the 24 h prior to their study visit to pick up the next set of crackers. At the end of each week, participants turned in a stool sample, as well as a completed questionnaire containing information regarding sample collection conditions, health, a diet checklist, and a 24-h dietary recall. Stool samples were immediately aliquoted and stored at −80 °C upon receipt by the lab. This study was approved by the Michigan State University Institutional Review Board (IRB #00002638).

### 2.2. Sample

A total of 33 participants were recruited using the Michigan State University paid research pool and flyers. Of the original sample, five were lost to follow-up in the run-in week. Hence, 28 participants provided fecal samples and data for analysis. The inclusion and exclusion criteria were described previously [24]. Briefly, participants had to be available for weekly lab visits, be between the ages of 18 and 55 years, have bowel movements at least once every three days, and be willing to eat wheat crackers. Participants were excluded if they reported taking any nutritional supplements, antacids, non-steroidal anti-inflammatory drugs (NSAIDs), proton pump inhibitors, or multivitamins on a daily basis; took antibiotics in the two weeks prior to or during the study; were pregnant; had gastrointestinal issues or diabetes; had food allergies; or followed a special diet.

### 2.3. Measurement

#### 2.3.1. Participant Characteristics

Participants provided demographic and health information in an enrollment survey. This form collected information on age, height/weight, sex, antibiotic use, indicators of socioeconomic status, educational attainment, and race, among others (Table 2). At the end of each week, a food frequency questionnaire and 24-h recall with diet checklist were administered. This allowed for the calculation of individual dietary diversity scores following the Food and Agriculture Organization (FAO) guidelines [25], which enabled tracking average dietary diversity consistency during the study period. Diet diversity scores were not included in statistical models analyzing the gut microbiota diversity or composition. Moreover, the PhenX fiber intake protocol was used to assess participants’ daily fiber intake aside from the wheat crackers during the study. The protocol allowed participants to report how often they consumed a fiber-containing food: never, less than once a day, once a day, twice a day, three times a day, four times a day, or five or more times a day. Based on these reports, the PhenX protocol assigned scores to each participant that regarded for his/her fiber intake outside of the assigned wheat crackers during each week. Participants also indicated the estimated percentage of crackers eaten in the past week using a 100 mm scale labelled at 0%, 50%, and 100%. We then visually assigned the participants’ estimation to 0%, 25%, 50%, 75%, or 100% compliance levels.

#### 2.3.2. Microbiota Measurement

The procedures to extract DNA from fecal samples, amplify the V4 region of the 16S rRNA gene, and sequence those amplicons were previously described [26]. Processing of sequence reads occurred in mothur [27], using the Michigan State University High Performance Computing Cluster. Sequences were rarified to 9999 reads per sample (performed 999 times, averaged, and then rounded), and rarefaction curves confirmed adequate community coverage.

Alpha (within participant) and beta (between participants by cracker type consumed) diversity indices were used to characterize the gut microbial diversity. We quantified alpha diversity using three metrics, Chao1, Shannon, and Inverse Simpson. Each provides insight into the overall community diversity within an individual by accounting for richness (Chao1) or both richness and evenness (Shannon/Inverse Simpson). A higher score indicates a higher diversity. Beta diversity was visualized by plotting the Sorensen and Bray–Curtis dissimilarity scores on a principal coordinates plot. An individual is represented by a single point, and the closer two points are to each other, the more similar the respective gut microbial communities of those two individuals. Clustering of samples by timepoint would suggest differences in community composition by wheat treatment.

#### 2.3.3. Intestinal Inflammation Measurement

Intestinal inflammation was determined by examining both the presence of inflammatory proteins in stool and the abundance of butyrate-producing genes. The processes for each were previously described [24]. In brief, the intestinal protein markers of inflammation were calprotectin and lipocalin, which are reliable biomarkers for diagnosing/monitoring gastrointestinal inflammatory conditions such as inflammatory bowel disease [28]. Following extraction from the fecal samples, the concentrations of the proteins were determined using enzyme-linked immunosorbent assays (ELISAs).

Butyrate is an anti-inflammatory short-chain fatty acid (SCFA) produced by numerous commensals through two distinct metabolic pathways including the enzymes butyrate kinase and butyryl-CoA:acetate CoA-transferase. The abundance of these genes was quantified using quantitative real-time polymerase chain reaction (PCR) with primers designed by Vital and colleagues [29]. The higher the gene abundance, the greater the putative butyrate-production capacity and the lower the expected gastrointestinal inflammation.

### 2.4. Data Collection

Participants were enrolled in the summer of 2019, providing written informed consent at the time of enrollment. Study staff were on site to answer any questions and explain all stipulations for participating in the study. Participants were instructed to come to the laboratory at the start of each week and pick up their crackers. Then, at the end of the 7-day treatment period, while picking up the next set of crackers, participants would drop off the weekly fecal samples that they had collected at home with the use of fecal sample collection kits provided to them. These collection kits included Para-Pak Clean Vial tubes (Meridian, Biosciences, Cincinnati, OH, USA), which are where the stool samples were stored at room temperature until drop-off. After drop-off, stool was aliquoted and stored at −80 °C until further analysis. In total, four sets of crackers were provided to each participant, and four fecal samples per participant were provided to the laboratory.

### 2.5. Data Analysis

All statistical analyses were conducted in R version 4.0.2 [30]. Alpha and beta diversity scores were calculated with the vegan package [31]. A Friedman test was used to test for differences between study timepoints for alpha diversity. Beta diversity dissimilarity scores were ordinated using a principal coordinates analysis, and permutational multivariate analysis of variance was used to test for differences in beta diversity between study timepoints. A negative binomial mixed model from the NBZIMM package was used to test for changes in average relative abundances of taxa by timepoint [32]. A Friedman test was also used to test for differences in butyrate-producing gene counts by timepoint. Due to missing data, comparisons between paired control (run-in/washout) and treatment timepoints were conducted using the paired Wilcoxon test for concentrations of inflammatory proteins. Fiber analysis included calculating median and mean fiber intake values across all participants for each timepoint. Outliers were considered to be any value 50% greater than the 75th percentile, and 50% lower than the 25th percentile of the overall participant fiber intake data for each week, and these values were omitted from calculations.

## 3. Results

### 3.1. Participant Characteristics

Of the participants (n = 28), 60.7% (n = 17) were female and 85.7% (n = 24) were college graduates (Table 2). Throughout the study, participants consumed greater than 80% of their crackers, on average. Most participants were White, non-Hispanic (60.7%; n = 17), and about half were earning an annual income of less than USD 25,000 (51.9%; n = 14). The average age of the participants was 35.2 years, and the average BMI was 26.0.

Diet is a major factor shaping the gut microbiome, as well as the abundance of various microbially derived metabolites, such as SCFAs. To that effect, it was important that we tracked dietary patterns among our sample of free-living adults. To quantify this, we utilized the dietary diversity score developed by the FAO, a tool designed to score and analyze dietary diversity using dietary questionnaire responses [25]. Although participants were free to eat what they chose in addition to the allotted daily proportion of crackers, the average dietary diversity score was consistent across timepoints, with changes of less than one point out of 10 on average (Appendix A). In microbiome-focused dietary intervention studies, the stabilization, rather than standardization, of diets is recommended [33]. As such, participants were encouraged to maintain their normal diets aside from the wheat intervention. However, changes in dietary diversity scores indicated that individual dietary intake by participants varied over time. The difference between diet diversity scores for each participant was, on average, 2, and the median was a difference of 1 unit. Further, individuals consumed diets with widely varying diet diversity at each timepoint. Another method by which the diets of participants were measured was by analyzing mean fiber consumption (in grams per day) across all participants throughout the study. With outliers omitted, the mean and standard deviation of fiber consumption for Weeks A, B, C, and D were evaluated to be 21.7 ± 8.1 g/day, 18.6 ±5.9 g/day, 17.1 ± 3.9 g/day, and 18.1 ± 5.5 g/day, respectively.

### 3.2. Gut Microbiota Diversity

**Figure 1:**
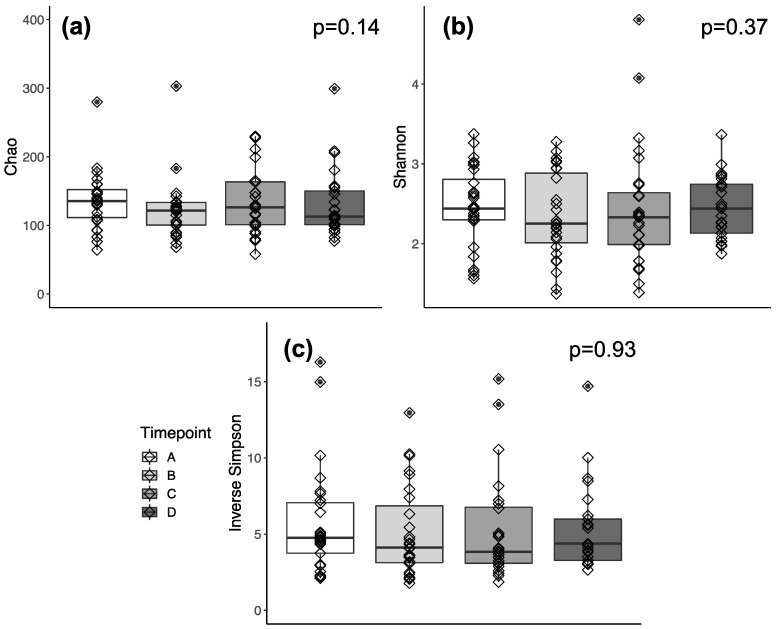
(**a**) Chao, (**b**) Shannon, and (**c**) Inverse Simpson indices for alpha diversity in stool samples (*p* > 0.05; Friedman X^2^) across the weekly timepoints (A, B, C, and D). Note: For purposes of graphical scale, three outliers are not shown in (**a**). One outlier is not shown in (**b**). Outliers are defined as values falling beyond 1.5 × IQR + Q3 or Q1 − 1.5 × IQR (the whiskers). The center line of each box is the median value, and the top and bottom edges are Q3 and Q1, respectively. IQR, Interquartile Range; Q3, Third Quartile; Q1, First Quartile. Timepoints A, B, C, and D refer to the 7th day of eating wheat crackers made from the following: Week A, refined soft white wheat flour; Week B, whole-grain soft white wheat flour; Week C, refined soft white wheat flour; Week D, whole-grain soft red wheat flour.

**Figure 2:**
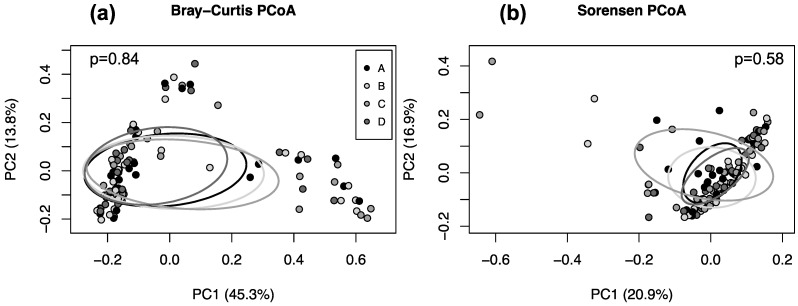
Principal coordinate plots of the (**a**) Bray–Curtis and (**b**) Sorensen dissimilarity scores of fecal bacterial communities in stool samples collected from participants consuming wheat-cracker treatments. Note: The percentage of variation accounted for by each axis is shown. Ellipses depict the standard deviation of points from the centroid of each timepoint. PC1, Principal Coordinates Axis 1; PC2, Principal Coordinates Axis 2. Timepoints A, B, C, and D refer to the 7th day of eating wheat crackers made from the following: Week A, refined soft white wheat flour; Week B, whole-grain soft white wheat flour; Week C, refined soft white wheat flour; and Week D, whole-grain soft red wheat flour.

The alpha (Figure 1) and beta (Figure 2) diversities of the gut microbiotas of participants were similar across timepoints, regardless of diversity metric used.

### 3.3. Gut Microbiota Taxa

**Figure 3:**
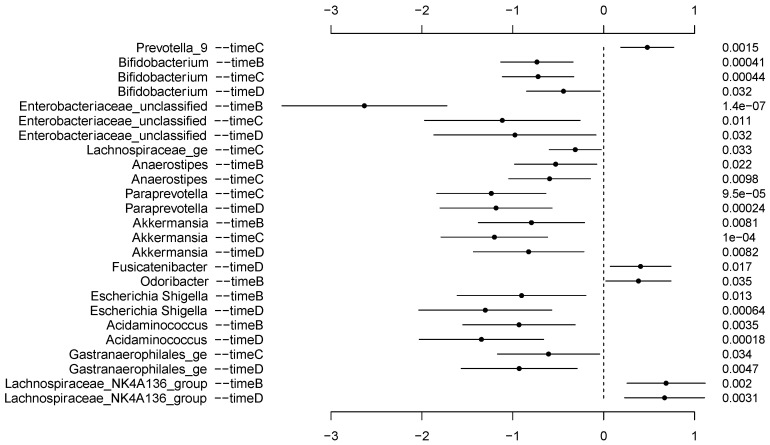
Differences in average relative abundances of taxa in the stool bacterial microbiotas of participants consuming the wheat cracker treatments for each week. The reference timepoint is Week A. Note: The negative binomial regression coefficient and 95% confidence interval band is shown for each taxon/timepoint. The *p*-values are listed in the column to the right. Only taxa with greater than 0.5% average relative abundance were included in this analysis. Timepoints A, B, C, and D refer to the 7th day of eating wheat crackers made from the following: Week A, refined soft white wheat flour; Week B, whole-grain soft white wheat flour; Week C, refined soft white wheat flour; and Week D, whole-grain soft red wheat flour.

When compared to the reference run-in period, numerous taxa were enriched or depleted at the treatment and washout timepoints (Figure 3). Notably, *Bifidobacterium*, a taxon of interest due to its probiotic potential, was significantly decreased at all timepoints relative to the run-in (Week A). *Enterobacteriaceae* unclassified and *Akkermansia* were also significantly depleted at all study timepoints compared to the run-in.

Only four taxa were enriched following Week A. These were *Odoribacter* in Week B, *Prevotella* 9 in Week C, and *Fusicatenibacter* in Week D. *Lachnospiraceae NK4A136* group was enriched during both whole-grain weeks (Weeks B and D). Conversely, *Acidaminococcus* and *Escherichia*/*Shigella* were depleted during the whole-grain weeks.

### 3.4. Gastrointestinal Inflammation

#### 3.4.1. Inflammatory Protein Markers

In addition to analyzing the impact of the wheat cracker intervention on gastrointestinal bacterial communities, we also analyzed its impact on markers of gastrointestinal inflammation. Two established protein biomarkers of intestinal inflammation are calprotectin and lipocalin-2, and these proteins were extracted from submitted stool samples to determine whether the whole-grain intervention-induced anti-inflammatory effects among a sample of healthy participants. However, at the cracker dose provided, neither whole-grain nor RSW wheat-flour types had a significant impact on levels of biomarkers of intestinal inflammation, as all levels remained steady throughout the duration of the study. The concentrations of lipocalin and calprotectin were similar across timepoints (*p* > 0.05; paired W) (Table 3).

#### 3.4.2. Butyrate Production Capacity

The gut microbiota has been hypothesized to exert its beneficial effects through the associated metabolites that it produces. Many of these metabolites are related to the nutrients that are available to the bacterial species present within the gut. One notable example is the production of SCFAs from fermentable fibers. In this study, the abundances of butyrate-producing genes were similar across timepoints (*p* > 0.05; Friedman X2) (Table 4).

## 4. Discussion

Consumption of different whole-grain wheat flour types for a single-week duration per type, and at a daily dose of 80 g of wheat flour crackers, had a minimal impact on gut microbial diversity and intestinal inflammatory biomarker levels. However, the abundance of several microbial taxa changed during the study period.

### 4.1. Gut Microbiota Diversity

The alpha (Figure 1) and beta (Figure 2) diversities of the gut microbiotas of participants were similar across timepoints, regardless of diversity metric used. Alpha diversity provides insight into the overall gut microbial diversity within a participant. The lack of significant differences suggests that soft wheat flour, regardless of the type, has little effect on the gut microbiome of free-living, healthy adults at the provided dose. This is further supported by two human trials that observed no change in alpha diversity after two 8-week periods or a 6-week period, respectively, of a mixed whole-grain intervention [34,35]. These findings indicate that wheat and its constituents, such as fiber, do not influence the gut microbiota alpha diversity in the healthy adults studied.

Nonetheless, it is important to acknowledge research demonstrating that fiber-rich diets, which often contain wheat products such as whole-grain bread, are frequently associated with higher gastrointestinal microbiota alpha diversity [36,37]. However, the studies that report this are usually examining effects of overall diet rather than a single constituent, such as fiber. Specifically, they tend to be observational and identify a link between fiber-rich foods, rather than the fiber alone, and microbial diversity. Additionally, the response to fiber-rich foods is individualized and likely to differ substantially across individuals [38]. Those that examine the effects of fiber alone report little change in alpha diversity following fiber interventions, which would further support the observed similarities in alpha diversity across timepoints in this wheat cracker intervention study [39,40]. Other studies demonstrate that phenolic compounds of wheat have strong anti-inflammatory effects and promote gastrointestinal health [41]. Hence, we predicted that constituents of wheat as a whole, rather than fiber alone, may be more consequential in modulating the alpha diversity of the gut microbiome.

Studies that aim to identify fiber effects on the gut microbiota often use fixed diets, which are not realistic among free-living adults [42]. Moreover, if model organisms are used instead of humans, it becomes difficult to generalize the results across species due to differences in microbial communities and metabolic pathways, as well as dose differences [43]. Finally, it is exceedingly complex to identify the effects of a single dietary constituent on the gut microbiota due to underlying genetic and lifestyle differences between participants that could confound the observed associations [44]. As such, research on the individualized responses of the gut microbiota to fiber intake in free-living humans is still underdeveloped.

Beta diversity provides insight into differences in gut microbial composition between samples. Like alpha diversity, there were no significant differences in beta diversity by treatment week (Figure 2). This agrees with prior research examining the effects of wheat-related interventions on measures of overall gut microbial diversity. For instance, one group reported minimal change in beta diversity following two-week treatment periods with high (28 g) and low (14 g) levels of fiber from whole-wheat/bran cereal [45]. Another study conducted a 12-week whole-grain vs. refined-wheat intervention, with participants consuming 98 g of wheat per day, and found no significant changes in beta diversity [46]. Together, these findings suggest that whole wheat consumption does not alter beta diversity in freely living humans.

### 4.2. Gut Microbiota Taxa

Although the wheat interventions of this and some prior studies have seemingly little effect on overall gut microbial diversity, these types of dietary interventions have been observed to induce changes in the relative average abundances of various taxa. Notably, *Bifidobacterium*, a taxon of interest due to its probiotic potential, was significantly decreased at all timepoints relative to the run-in (Week A). This contradicts previous research where a bifidogenic effect of whole-grain wheat was observed [47,48]. *Enterobacteriaceae* unclassified and *Akkermansia*, the latter being a beneficial genus that has been implicated in maintaining the gut barrier [49], were also significantly depleted at all study timepoints compared to the run-in.

Only four taxa were enriched following Week A. These were *Odoribacter* in Week B, *Prevotella* 9 in Week C, and *Fusicatenibacter* in Week D. The *Lachnospiraceae NK4A136* group was enriched during both whole-grain weeks (Weeks B and D). This agrees with previously published work showing that members of *Lachnospiraceae* are positively associated with wheat/grain-based foods and fiber-rich diets [40,45,50]. Conversely, *Acidaminococcus* and *Escherichia*/*Shigella* were depleted during the whole-grain weeks. *Escherichia*/*Shigella* are commonly associated with disease [51]. A previous study showed that Enterobacteriaceae, the family to which *Escherichia/Shigella* belong, was also depleted following whole-grain consumption [35]. This suggests a potential protective effect of whole wheat on gut health by decreasing the likelihood of overgrowth of these opportunistic pathogenic taxa.

### 4.3. Gastrointestinal Inflammation

#### 4.3.1. Inflammatory Protein Markers

In addition to analyzing the impact of the wheat cracker intervention on gastrointestinal bacterial communities, we also analyzed its impact on markers of gastrointestinal inflammation. This is timely considering the emerging science on the potential anti-inflammatory effects of whole-grain intake, especially among unhealthy participants, including those with diabetes, hyperlipidemia, gastrointestinal disease, cardiovascular disease, or other chronic diseases [17]. Two established protein biomarkers of intestinal inflammation are calprotectin and lipocalin-2 [28,52]. We extracted these proteins from submitted stool samples to determine whether the whole-grain intervention induced anti-inflammatory effects among a sample of healthy participants. However, at the cracker dose provided, neither whole-grain nor RSW wheat-flour types had a significant impact on levels of biomarkers of intestinal inflammation, as all levels remained steady throughout the duration of the study. The concentration of lipocalin and calprotectin were similar across timepoints (*p* > 0.05; paired W) (Table 2). Hence, the beneficial health effects of wheat may not be as prominent in healthy, free-living individuals who have low levels of basal inflammation. This finding is consistent with those for healthy participants who are slightly overweight or when different biomarkers of inflammation are measured [53,54]. Overall, in spite of the epidemiological evidence suggesting benefits of wheat consumption, intervention trials in healthy populations are still inconclusive regarding the exact impacts of wheat consumption on gastrointestinal inflammation [17,55,56].

#### 4.3.2. Butyrate Production Capacity

The gut microbiota has been hypothesized to exert its beneficial effects through the associated metabolites that it produces. Many of these metabolites are related to the nutrients that are available to the bacterial species present within the gut. One notable example is the production of SCFAs from fermentable fibers. It has been established for some time that SCFAs have beneficial health effects, including improvement of gut barrier function, as well as decreasing inflammation [57]. Butyrate specifically is a major anti-inflammatory SCFA, and many of the microorganisms that produce butyrate are considered to be probiotics [58]. Thus, the beneficial health effects of whole-grain wheat could be derived from the increase in associated SCFA production [59]. For this reason, we measured the abundance of the bacterial genes required to produce butyrate. It was expected that this butyrate production capacity would increase with whole-grain wheat treatments. Measuring the capacity of bacteria for butyrate production has been reliably associated with fecal butyrate levels and is therefore a valid proxy [60].

The similarities in butyrate production-gene abundances observed week by week in this intervention trial suggests there is minimal impact on the putative capacity for butyrate production as a result of the wheat cracker interventions with the provided flour types and doses. A prior study also reported minimal changes in the abundance of butyrate-producing genes following an eight-week, mixed whole-grain diet [34]. In overweight/obese adults, SCFA gene counts did not differ after a 12-week whole-grain wheat intervention, except for butyrate kinase [46]. Hence, the effect of whole-grain wheat on butyrate remains unclear. Nonetheless, this does not exclude the possibility that wheat does decrease overall inflammation through pathways other than those in which butyrate is involved.

### 4.4. Strengths, Limitations, and Other Considerations

This study demonstrates possible differences in effects of ingested wheat flour types on the gut microbiome and intestinal inflammatory markers. Its strengths include the provision of realistic and manageable doses of wheat flour crackers to a free-living adult population, as well as the longitudinal study design. Many other studies examine changes in habitual consumption that extend for long periods of time or entail large consumption of whole grains that any effects observed may be unsustainable in a free-living population. Another strength of this study is its inclusion of various markers of intestinal inflammation which provides broader insight into the intestinal state.

Nonetheless, it is prudent to note the limitations of this work when interpreting the results and implications. First, the sample size was limited, which decreases the ability to generalize our results. Larger sample sizes would enable stratified analyses based on each participant’s starting gut microbiota composition, which could improve detection of taxa-level microbiota shifts. However, this research contributes important information to the body of literature on this topic because it provides details about the study design, as well as the results of the intervention, so that others can learn from the results to design improved studies in the future. The analysis does not consider the effect of other heath conditions of participants, other than exposure to antibiotics. Additionally, since participants comprised of a general, healthy population of freely living adult humans, there is a decreased likelihood of observing uniform changes in the gut microbiota secondary to wheat cracker intervention. A specific, targeted population, such as participants with conditions like diabetes or rheumatoid arthritis, may have gut microbiotas that are more prone to change as a result of such interventions [61]. Hence, more definitive results may have been produced when assessing changes in abundance of butyrate production genes or intestinal inflammation biomarker concentrations if such populations were used. The collection of serum samples in order to assess systemic levels of inflammation, through measurement of cytokines or soluble CD-14, would have been informative and could improve understanding of the impact of the intervention on inflammation. Unfortunately, serum samples were not collected.

A limitation to the habitual diet design of this study is that we cannot know whether the obtained data resulted from the designed dietary intervention or from diversity in an individual’s diet. Future work should focus on precision analysis of individual-level data. It is important to note that the observed stability of the dietary diversity scores may indicate that the overall diet was stable and that differences in the gut microbiome or inflammatory markers arose due to the intervention rather than fluctuating dietary habits. However, this is impossible to fully disentangle given the study design employed herein. Hence, overall dietary intake is a key confounder to acknowledge. Since all participants consumed the same crackers each week, it is difficult to discern whether changes in the gut microbiome were due to the crackers alone. A crossover design that randomized the order in which cracker types were consumed would be more robust. Moreover, with the participants free to consume as many or as few of the crackers as they pleased, not all participants consumed equal levels of crackers at each timepoint. Nonetheless, overall compliance remained high (>80%) throughout the study. Additionally, it is crucial for researchers to take into consideration how a dietary intervention could influence participants’ overall intake of the supplement focused on by the intervention. In this study, it was observed that participants’ median and mean fiber intakes (in grams per day) conveyed an overall negative trend from Week A to Week D. This can be explained from a psychological standpoint; when participants are given wheat crackers to eat, they could subconsciously or consciously deem this intervention as substituting their daily fiber intake rather than adding to it. As a result, participants might have chosen to omit the fiber-rich foods that they would have consumed otherwise, decreasing their overall fiber intake and lowering the likelihood of detecting changes in the gut microbiome. Therefore, it is essential for researchers to inform and encourage participants to approach a dietary intervention as being a supplement to, rather than a substitution for, their existing diet.

It would also be beneficial for future researchers to ensure adequate fecal material for both metagenomic and ELISA analyses. In this study, the missing data points for calprotectin/lipocalin levels restricted our analysis to pairwise, rather than longitudinal, comparisons. Additionally, the differences in taxa level by timepoint are correlational. Finally, the fecal samples were exposed to room temperature prior to delivery to the laboratory [62]. Due to the resulting high moisture content of fecal samples, the obtained results of the microbial composition may have been altered, but the results reported retain their validity because any changes induced are conserved across all samples [63,64].

## 5. Conclusions

Our study is the first to examine potential differences in health effects of consuming 80 g daily of wheat flour crackers, made from various types of wheat flour, on the gut microbiome and inflammatory markers of a free-living adult population. The abundance of some bacterial taxa, including *Escherichia/Shigella, Acidaminococcus*, and the *Lachnospiraceae NK4A136* group, were altered with whole-grain wheat intervention, but overall microbial diversity and markers of intestinal inflammation were unchanged. This suggests that the beneficial health effects of the consumption of crackers made from whole-grain wheat flour may depend on specific members of the gut microbiota. The specific type of wheat (soft white or soft red) was not associated with microbiome diversity or inflammatory outcomes. Additionally, the seven-day intervention may have been too short to induce significant changes in the gut microbiota or inflammatory markers. Further research is needed to identify the exact mechanisms underlying the potential health benefits of whole-grain wheat and to determine which, if any, of these mechanisms are microbially related.

## Figures and Tables

**Table 1:** Nutrient composition of 80 g of test cracker by cracker type.

	Calories (kcal)	Fat (g)	Saturated Fat	MUFAs ^a^	PUFAs ^b^	Trans Fat	Protein (g)	Total Sugars (g)	Other Carbohydrates (g)	Total Dietary Fiber (g)	Insoluble Fiber (g)	Soluble Fiber (g)	Sodium (mg)	Ash (g)	Moisture (g)
Refined soft white wheat	313	6.44	1.68	1.26	3.16	0.058	7.15	2.44	54.12	3.99	1.54	2.45	439	1.512	4.35
Whole-grain soft white wheat	298	7.16	1.82	1.41	3.54	0.071	9.1	2.816	46.65	10.72	7.54	3.18	452	2.112	1.44
Whole-grain soft red wheat	306	8.00	2.08	1.59	3.92	0.082	9.35	2.392	46.61	10.72	7.35	3.37	454	2.152	0.78

^a^ MUFAs: monounsaturated fatty acids. ^b^ PUFAs: polyunsaturated fatty acids.

**Table 2:** Participants’ characteristics.

Characteristic	Mean	SD
BMI	26.0	5.7
Age ^1^ (years)	35.2	9.9
Compliance ^2^ (% consumed)		
Week A ^3^	89.4	17.6
Week B ^1^	84.3	19.8
Week C ^1^	82.4	16.7
Week D ^3^	80.8	26.7
	*n* (N = 28)	%
Female	17	60.7
Ever smoked	7	25.0
Antibiotics in the past year	11	39.3
Homeowner	10	35.7
Car owner ^3^	20	76.9
Stocks/bonds owner ^3^	10	38.5
BMI Category		
Underweight	1	3.6
Normal	12	42.9
Overweight	9	32.1
Obese	6	21.4
Race		
White—non-Hispanic	17	60.7
White—Hispanic	2	7.14
Black/African American	2	7.14
Other	7	25.0
Education		
College graduate or more	24	85.7
Some college	4	14.3
Income ^1^		
Under USD 25,000	14	51.9
USD 25,000–USD 49,000	1	3.7
USD 50,000–USD 75,000	8	29.6
Over USD 75,000	4	14.8

^1^ One missing data point. ^2^ Percent of crackers consumed by each participant averaged per timepoint. ^3^ Two missing data points. SD, standard deviation; BMI, body mass index. Crackers consumed were made from: Week A, refined soft white wheat flour; Week B, whole-grain soft white wheat flour; Week C, refined soft white wheat flour; and Week D, whole-grain soft red wheat flour.

**Table 3:** Median and range of lipocalin and calprotectin protein concentrations (ng/mL extraction buffer) in fecal samples collected after participants ate the treatment crackers for each week.

Timepoint	Lipocalin	Calprotectin
Week	Median (Range)	Median (Range)
A ^1^	21.1 (0.7–192.4)	46.8 (5.0–1915.1)
B ^2^	14.8 (0.5–64.8)	46.6 (3.1–1754.3)
C ^3^	21.7 (2.7–146.0)	33.2 (5.1–1888.3)
D ^4^	22.6 (1.4–71.8)	62.5 (8.0–1895.0)

^1^ Two missing data points. ^2^ Five missing data points. ^3^ Eight missing data points. ^4^ Six missing data points. Note: Data points were missing because some fecal samples were not sufficient for both microbiota analysis and inflammatory marker analysis. Crackers consumed were made from the following: Week A, refined soft white wheat flour; Week B, whole-grain soft white wheat flour; Week C, refined soft white wheat flour; and Week D, whole-grain soft red wheat flour.

**Table 4:** Median and range of butyrate-producing gene abundances as quantified by quantitative real-time PCR of genomic DNA isolated from fecal samples obtained after each week of cracker consumption.

	Week A	Week B	Week C	Week D	Friedman X^2^	*p*-Value
Buk ^1^	25.0 (0–114.0)	25.5 (0–210.0)	27.0 (1.0–341.0)	26.0 (0–113.0)	1.91	0.59
ButFPrausn ^2^	24,074 (2105–141,161)	32,115 (22–135,793)	28,970 (154–78,285)	40,029 (6150–219,352)	1.98	0.58
ButRosEub ^2^	510,396 (59,266–3,239,517)	696,317 (1905–4,018,413)	470,724 (373–4,185,708)	432,130 (4405–4,174,740)	4.56	0.21

^1^ One missing data point at Week A. Two missing data points at Week B. ^2^ One missing data point at Week B. Crackers consumed were made from the following: Week A, refined soft white wheat flour; Week B, whole-grain soft white wheat flour; Week C, refined soft white wheat flour; and Week D, whole-grain soft red wheat flour. Note: Buk primers are based on the butyrate kinase gene. ButFPrausn primers are based on the sequence of the butyryl-CoA:acetate CoA-transferase gene in *Faecalibacterium prausnitizii*. ButRosEub primers are based on the sequence of the butyryl-CoA:acetate CoA-transferase gene in *Roseburia* and *Eubacterium* spp.

**Figure A1:**
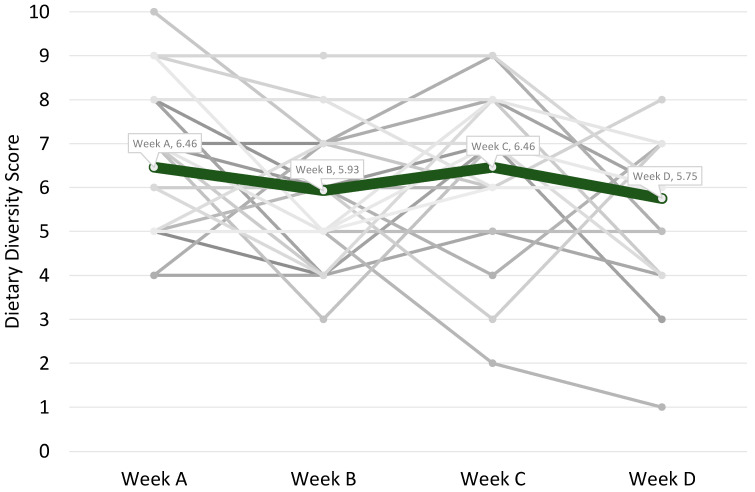
Food and Agriculture Organization dietary diversity scores across study timepoints. Thin lines are values for each participant. The thicker line is the mean value. Note: One missing data point in Week B. Timepoints A, B, C, and D refer to the 7th day of eating wheat crackers made from the following: Week A, refined soft white wheat flour; Week B, whole-grain soft white wheat flour; Week C, refined soft white wheat flour; Week D, whole-grain soft red wheat flour.

## Data Availability

Data are available from the corresponding author. The data are not publicly available due to the small sample size and restrictions of participant consent forms.
